# Determining Maximum Glycolytic Capacity Using Extracellular Flux Measurements

**DOI:** 10.1371/journal.pone.0152016

**Published:** 2016-03-31

**Authors:** Shona A. Mookerjee, David G. Nicholls, Martin D. Brand

**Affiliations:** 1 Touro University California College of Pharmacy, 1310 Club Drive, Vallejo, California, 94592, United States of America; 2 Buck Institute for Research on Aging, 8001 Redwood Blvd, Novato, California, 94945, United States of America; University of Alabama at Birmingham, UNITED STATES

## Abstract

Measurements of glycolytic rate and maximum glycolytic capacity using extracellular flux analysis can give crucial information about cell status and phenotype during normal operation, development of pathology, differentiation, and malignant transformation. They are also of great use when assessing the effects of chemical or drug treatments. Here, we experimentally define maximum glycolytic capacity, demonstrate how it differs from glycolytic rate, and provide a protocol for determining the basal glycolytic rate and maximum glycolytic capacity in cells using extracellular flux measurements. The results illustrate the power of extracellular flux analysis to describe the energetics of adherent cells in culture in a fully quantitative way.

## Introduction

Biological systems generally operate at a metabolic rate that is lower than the highest rate achievable, allowing them metabolic scope to respond to changing demands. The maximum rate is referred to as “metabolic capacity”, and constrains the response a cell can have to acute increases in energy demand. In most cells, the metabolic rate is largely determined by the current energy demand, and within seconds it responds quantitatively and sensitively to changes in that demand over a wide range. Metabolic capacity is plastic over longer timeframes of hours to days, as cells adjust to altered or anticipated demand by synthesis or degradation of their enzymatic machinery. Inappropriate decreases in metabolic capacity impair the matching of supply to demand and are associated with multiple pathologies and aging-related dysfunction (for recent reviews, see [1–3]).

There are two major components of metabolic capacity, respiratory and glycolytic. Although full flux analysis using tracers can be used to quantify them, it is often more convenient to distinguish and measure these components by the rates of change in extracellular concentrations of dissolved oxygen (O_2_) and protons (H^+^), respectively.

Respiratory capacity is a measure of the maximum rate of substrate catabolism and mitochondrial electron transport (and hence O_2_ consumption) that can be achieved acutely by a cell. It is often equated to the maximum rate of oxidative phosphorylation, but since electron transport can be uncoupled from ATP synthesis, this is not always appropriate; in cells with limited ATP synthase activity (such as brown adipocytes) respiratory capacity can exceed the capacity for oxidative phosphorylation several-fold. Respiratory capacity can be experimentally defined and quantitatively measured as the mitochondrial oxygen consumption rate during optimal uncoupling (to avoid any limitation by the coupled rate of ATP synthesis) [4].

Glycolytic capacity is a measure of the maximum rate of conversion of glucose to pyruvate or lactate that can be achieved acutely by a cell. Since glycolytic ATP synthesis is obligatorily linked to glycolytic carbon flux, glycolytic capacity is also a measure of the maximum capacity of glycolysis to generate ATP. Catabolism of one glucose to two lactate^-^ necessarily generates two H^+^ (which are exported with the lactate, maintaining cytosolic pH), therefore, glycolytic rate to lactate is measurable using the acidification of the extracellular medium. However, protons are generated during both glycolysis (by production of lactate^-^ + H^+^) and respiration (by production of CO_2_, which is converted to HCO_3_^-^ + H^+^). This ambiguity leads to a rate of total extracellular acidification that can be greater than glycolytic rate to lactate, because it is contaminated to varying degrees (ranging from 0 to 100%) by protons derived from respiratory CO_2_ production. We recently addressed this issue and developed a simple method for correcting the total extracellular acidification signal using oxygen consumption data, to isolate glycolytic acidification and therefore glycolytic rate [5, 6].

Glycolysis and glycolytic capacity are widely investigated in cellular models. Glycolysis is proposed to be the major ATP source for plasma membrane ion transporters in some cancer models [7]. Glycolytic capacity is proposed to be a predictor of drug sensitivity in tumor models [8, 9], and of immune tolerance in dendritic cell models [10]. It is also associated with cell damage; decline in apparent glycolytic capacity is observed during hyperoxia [11] and in a heart failure model [12]. Finally, increased apparent glycolytic capacity is associated with cellular reprogramming and differentiation [13, 14].

The experimental conditions that maximize glycolytic rate to lactate to allow estimation of maximum glycolytic capacity are not well defined. To date, conditions that starve the cell of all sources of ATP production save glycolysis are used to achieve this [15]. The major source of ATP in most cells is oxidative phosphorylation. Blocking this pathway with oligomycin (which inhibits the mitochondrial ATP synthase, preventing oxidative ATP production) shifts the burden of ATP supply entirely to glycolysis, markedly increasing glycolytic rate. In a cell with relatively limited glycolytic machinery, the resulting rate will be the maximum glycolytic capacity (unless the glycolytic capacity is so low that ATP levels fall below those needed to fuel hexokinase and phosphofructokinase, and glycolytic rate collapses; see Fig 5 in [16], and Fig 4 in [17]). However, in a cell with high glycolytic capacity, the glycolytic rate in the absence of oxidative phosphorylation may fully meet the whole of the cell’s current ATP demand without being maximal. To determine the maximum glycolytic capacity in such cells, in addition to isolating glycolysis as the sole ATP producer, cellular ATP demand must be increased until it just exceeds supply.

Here, we introduce and validate ways to increase ATP demand in cells sufficiently to cause maximum stimulation of glycolytic rate to lactate under conditions in which respiratory acidification of the medium are minimized. We demonstrate in myoblast and fibroblast cultures that the glycolytic rate elicited by oligomycin alone is significantly less than the maximum glycolytic capacity. This effect is obvious when ATP demand is artificially decreased by inhibition of protein synthesis, but is apparent even when it is not. We demonstrate that the glycolytic rate with oligomycin can be surpassed by replacement of oligomycin with rotenone and myxothiazol (to prevent oxidative phosphorylation, and, as added benefits, to fully prevent confounding respiratory acidification of the medium and to increase the rate of ATP hydrolysis by allowing reversal of the mitochondrial F_1_F_O_-ATP synthase). It is even greater following the further addition of monensin (to increase the import of Na^+^ into the cells and stimulate the rate of hydrolysis of ATP by the plasma membrane Na^+^/K^+^-ATPase). Finally, we describe a protocol for measurement of the basal glycolytic rate and maximum glycolytic capacity in cells.

## Materials and Methods

### Reagents

Chemicals were from Sigma. Cell culture reagents and consumables were from Corning. Seahorse XF24 consumables were from Seahorse Bioscience.

### Cells

Mouse C2C12 myoblasts were cultured under 95% air/5% CO_2_ in Dulbecco's modified Eagle medium (DMEM) with 11.1 mM glucose, 2 mM glutamine, 10% v/v fetal bovine serum (FBS), 100 U/mL penicillin and 100 μg/mL streptomycin. 24 h prior to assay, cells were plated in 100 μL culture medium at 20,000 cells/well in a 24-well polystyrene Seahorse V7-PS Flux plate with no additional coating. 25 min prior to assay, cells were washed three times with 500 μL Krebs Ringer Phosphate HEPES (KRPH) medium (2 mM HEPES, 136 mM NaCl, 2 mM NaH_2_PO_4_, 3.7 mM KCl, 1 mM MgCl_2_, 1.5 mM CaCl_2_, 0.1% w/v fatty-acid-free bovine serum albumin, pH 7.4 at 37°C) and kept at 37°C under 100% air. At assay start, medium was replaced with 500 μL KRPH containing 500 U/mL carbonic anhydrase (Sigma C2624). Two measurement cycles of 2 min mix, 1 min wait, and 5 min measure were carried out prior to addition of glucose, with either two or three measurement cycles following each subsequent addition.

HEK293 cells were grown and assayed identically as above, except that 10 mM HEPES was added to the DMEM cell culture medium described above. All cell lines were purchased or originally sourced from ATCC.

### Calculations

Separation of total extracellular acidification into respiratory proton production rate (PPR_resp_) and glycolytic proton production rate (PPR_glyc_) was carried out using Eq 1 as described in (5), with the same assumptions about substrate oxidation and substrate identity.

$$\begin{array}{l} \text{Glycolytic rate}={\text{PPR}}_{\text{glyc}}\\ \;=\left(\frac{{\text{ECAR}}_{\text{tot}}}{\text{BP}}\right)-({\text{OCR}}_{\text{tot}}-{\text{OCR}}_{\text{rot}/\text{myx}})(\text{max}\;{\text{H}}^{+}/{\text{O}}_{2})(\frac{10^{\text{pH}-{\text{pK}}_{1}}}{1+10^{\text{pH}-{\text{pK}}_{1}}}) \end{array}$$ Eq 1

where ECAR = extracellular acidification rate (mpH/min), tot = total, BP = buffering power (mpH/pmol H^+^), OCR = oxygen consumption rate (pmol O_2_/min), OCR_rot/myx_ = non-mitochondrial OCR remaining after complete inhibition of mitochondrial electron transport, max H^+^/O_2_ = the maximum H^+^ released to the medium per O_2_ consumed (and CO_2_ generated) by respiration, (see [5]), and K_1_ = the combined equilibrium constant of CO_2_ hydration and H_2_CO_3_ dissociation to HCO_3_^-^ + H^+^. The overall pK for CO_2(aq)_ + H_2_O → HCO_3_^-^ + H^+^ = 6.093 at 37°C ([18], p. 45). The spreadsheet used for these calculations in [6] incorporates Eq 1, enabling experimental data (starting pH, buffering power, maximum H^+^/O_2_, oxygen consumption rate, and total extracellular acidification rate) to be entered and proton production rate to be calculated. This spreadsheet is available for download [6].

For this calculation, we assumed that all of the CO_2_ produced remained in the XF24 wells [5], and that the cells used only the supplied glucose, which was completely oxidized. For complete oxidation of glucose, 1 CO_2_ is produced for each O_2_ consumed (i.e., the respiratory quotient, RQ, = 1), and a maximum of 1 H^+^ is generated by the hydration and dissociation of each CO_2_, giving a maximum H^+^/O_2_ ratio of 1. We assumed that prior to substrate addition the cells oxidized mixed endogenous substrates, primarily glycogen. Glycogen oxidation also has maximum H^+^/O_2_ of 1, and we therefore assumed an overall RQ of 1 and maximum H^+^/O_2_ ratio of 1 for pre-substrate-addition metabolism [5]. The separation of total extracellular acidification into respiratory and glycolytic proton production rates is accurate to the extent that these assumptions are correct; if, for example, substrate oxidation was incomplete and a significant fraction of the carbon was incorporated into molecules more reduced than CO_2_ (such as organic acids, proteins or nucleic acids), use of the maximum H^+^/O_2_ value would overestimate glycolytic rate. If pre-substrate-addition metabolism was primarily of substrates whose RQ is less than 1, such as fatty acids, using an RQ of 1 would underestimate glycolytic rate. However, these assumptions can easily be assessed for internal consistency by post-hoc measurement of lactate produced during the experiment; under the conditions used here for C2C12 myoblasts, measured lactate production agreed quantitatively with the amounts expected from calculated glycolytic rates after correction for respiratory proton production [5], suggesting that the assumptions were essentially correct.

### Statistical analysis

Data points are the mean of at least three independent biological replicates (see Fig legends) plotted with standard error of the mean. Comparisons within a given assay were done by one-way repeated measures ANOVA. Comparisons between assays were done by two-way repeated measures ANOVA. All ANOVA tests were followed by Bonferroni post-hoc multiple comparisons tests to determine significance. The mean of at least three technical replicates (i.e., three experimental wells) was used for each independent experimental point, but only the error between independent biological replicates was considered for statistical analysis.

## Results

### The conventional assessment of increased cellular dependence on glycolytic rate

**Fig 1:**
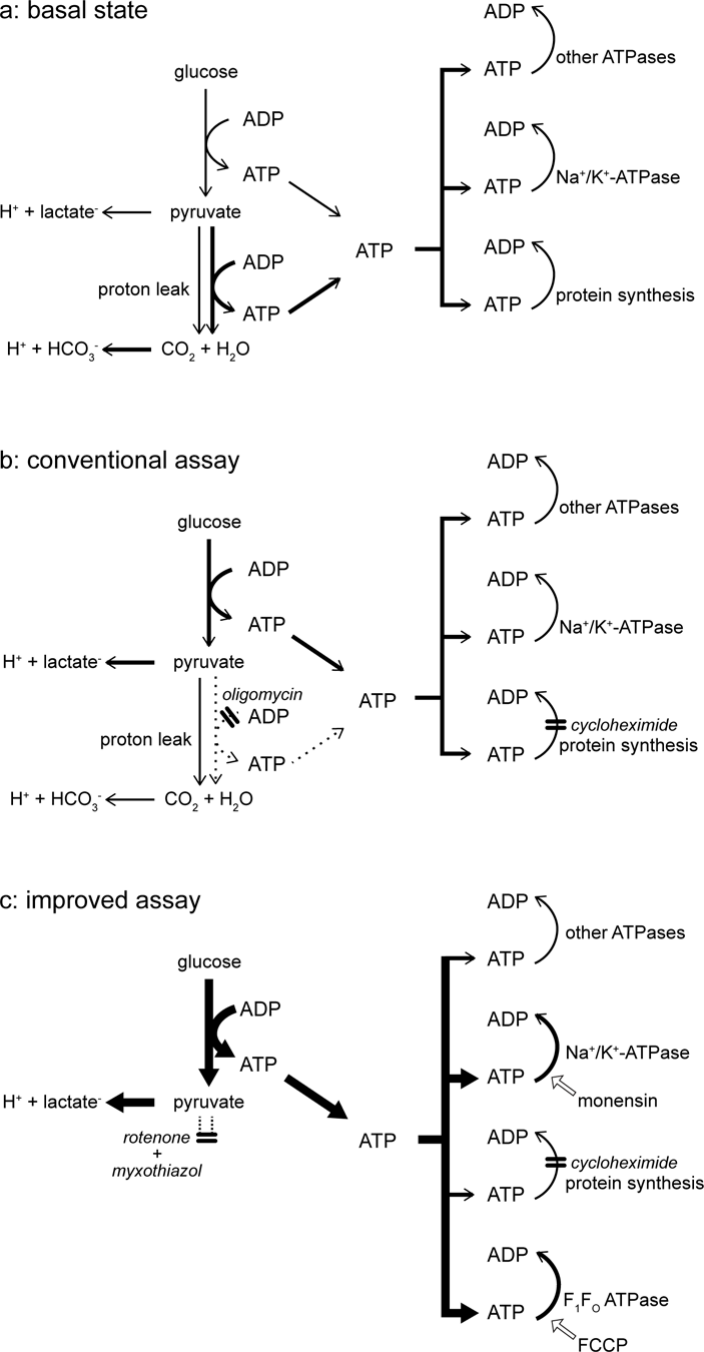
Pathways of H^+^ production and ATP production and consumption during measurement of glycolytic capacity. Relevant pathways under basal conditions (*a*), and during the conventional (*b*) or improved (*c*) assay of glycolytic capacity. Thicker arrows denote higher flux, dotted arrows denote zero flux. Double bars perpendicular to pathway arrows indicate sites of inhibition by the indicated compounds (cycloheximide was added only where indicated in later figures). Other additions are indicated with labelled, open block arrows. Only the protons associated with lactate^-^ and HCO_3_^-^, which contribute to extracellular acidification, are specified; the ionization states of pyruvate and adenine nucleotides are not shown.

Fig 1A shows the formal bioenergetics of a typical cell running on glucose. The rates of ATP production by glycolysis and oxidative phosphorylation are controlled primarily by ATP demand, represented here by protein synthesis, Na^+^/K^+^-ATPase and “other ATPases”. When the ATP demand by these ATP-consuming reactions is low, the rates of glycolysis and oxidative phosphorylation are low, and when ATP demand is high, glycolysis and oxidative phosphorylation run faster. The kinetics of different reactions within the cell determine the balance between ATP production by glycolysis-to-lactate on the one hand, and ATP production by the citric acid cycle and oxidative phosphorylation on the other, but in most aerobic cells ATP production is dominated by oxidative phosphorylation, as denoted by the heavier arrows in Fig 1A. Both reactions acidify the medium: partial oxidation of glucose to lactate^-^ is accompanied by stoichiometric production of H^+^, and complete oxidation of glucose to H_2_O + CO_2_ produces HCO_3_^-^ + H^+^. The total rate of extracellular acidification can be corrected for the rate of CO_2_ production (calculated from the rate of O_2_ consumption) to give the absolute rate of glycolysis-to-lactate [5].

Since glycolytic carbon flux is obligatorily coupled to glycolytic ATP production, to assess the maximum rate at which glycolysis can run (the glycolytic capacity), it is necessary to make ATP demand equal to or slightly greater than the capacity of glycolysis to supply ATP. This is most conveniently achieved when oxidative phosphorylation is prevented, forcing the cells to rely on glycolytic ATP production. The conventional way to do this is by addition of oligomycin, a specific inhibitor of the mitochondrial F_1_F_O_ ATP synthase. Fig 1B illustrates that when oxidative phosphorylation is blocked with oligomycin, demand is unchanged but ATP production shifts entirely to glycolysis.

**Fig 2:**
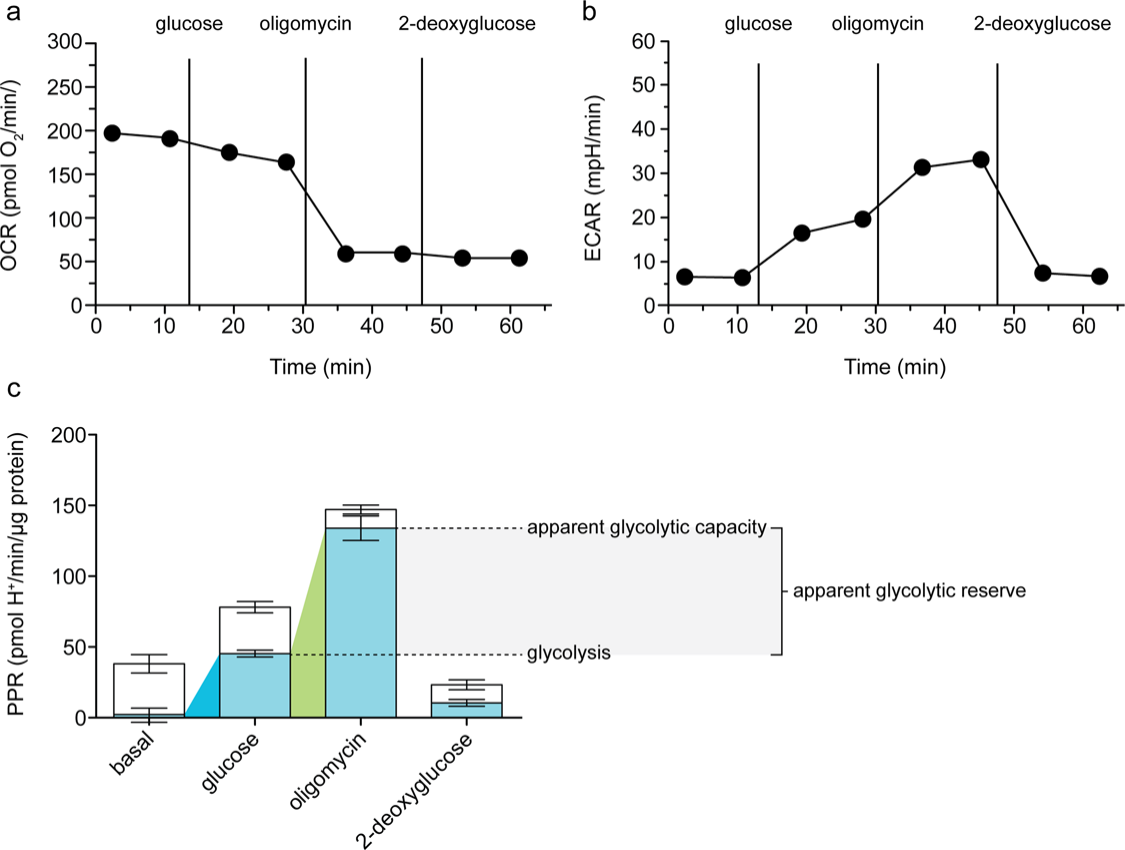
Increased glycolytic rate following inhibition of the F_1_F_O_-ATP synthase in C2C12 myoblasts. Raw traces of (*a*) oxygen consumption rate (OCR) and (*b*) extracellular acidification rate (ECAR) after sequential additions of 10 mM glucose, 2 μg/mL oligomycin, and 100 mM 2-deoxyglucose. One representative experiment is shown. *c*: Respiratory (open column sections) and glycolytic (blue column sections) proton production rates (PPR) of the experiment exemplified in a and b calculated using Eq 1. Coloured wedges indicate glycolysis under basal conditions (blue) and apparent glycolytic capacity (green), with the difference between these defined as apparent glycolytic reserve. Data are means ± SEM of n = 6 independent biological replicates.

Fig 2 shows the current standard experiment for increasing cellular dependence on glycolysis, run here in C2C12 myoblasts. In this experiment, extracellular flux of H^+^ is measured first in the absence of substrate, then after sequential additions of glucose to fuel glycolysis and respiration, oligomycin to inhibit the mitochondrial ATP synthase and respiratory ATP production (and therefore stimulate glycolytic flux), and 2-deoxyglucose to inhibit glucose catabolism. Fig 2A and 2B show the raw rates of oxygen consumption and extracellular acidification after each addition, and Fig 2C shows the calculated rates of proton production attributable to lactate production from glycolysis (in blue) and respiration (in white).

A conventional interpretation of this experiment is to consider the total proton production rate, shown in Fig 2B as raw data (rate of change of pH in the well), and in Fig 2C as the sum of respiratory and glycolytic rates (calibrated rates of glycolytic and respiratory H^+^ production normalized to cell number in the well). Addition of glucose increased the total proton production rate approximately 2-fold, from 38 to 78 pmol H^+^/min/μg protein (Fig 2C). Conventionally, this would imply a doubling of the glycolytic rate (or about a three-fold increase if the rate insensitive to 2-deoxyglucose is subtracted). Subsequent addition of oligomycin nearly doubled the rate again to 147 pmol H^+^/min/μg protein, because of the increase in glycolysis required to compensate for the loss of respiratory ATP production. Conventionally, the rate after oligomycin is interpreted to be the maximum glycolytic capacity. Finally, 2-deoxyglucose inhibited glycolysis and therefore decreased, but did not eliminate, total H^+^ production. Conventionally, the residual rate is interpreted to be non-glycolytic, and is therefore subtracted from all other rates.

There are several problems with this interpretation. First, it ignores respiratory acidification, and therefore overestimates the true glycolytic rate and underestimates the true magnitude of the changes in glycolytic rate. Second, it assumes that the rate after addition of 2-deoxyglucose should be subtracted from the other rates, which is hard to justify (see below). Third, it assumes that the rate of acidification in the presence of oligomycin is the maximum glycolytic capacity, which is not necessarily correct.

The first and second problems are overcome in Fig 2C by separating out the rate of acidification due to lactate production (blue bars) from the rate due to respiratory CO_2_ production (white bars) [5]. This correction reveals that the basal proton production rate was entirely respiratory, with negligible contributions from lactate production or background acidification or drift. Addition of glucose increased the glycolytic proton production rate to 45 pmol/min/μg protein, about 60% of the total rate. This was a tens- to hundreds-fold (or more) increase in glycolytic rate, rather than the 2-3-fold increase suggested by the conventional interpretation. Glucose addition did not increase respiratory proton production, suggesting that unspecified endogenous respiratory substrates were not limiting in the basal state. Instead, there was a small decrease in respiratory acidification rate. This was observed consistently (see later Figs) and was presumably caused by the compensatory decrease in the rate of oxidative phosphorylation when increased glycolytic rate driven by added glucose tended to raise the total rate of ATP synthesis at constant ATP demand. This is the Crabtree effect, in which ATP production following addition of a glycolytic substrate occurs preferentially through glycolysis rather than oxidative phosphorylation, and respiration can be repressed by as much as 50% (reviewed in [19]). By assuming reducing equivalent transfer into the mitochondrial matrix by the malate-aspartate shuttle, as well as no change in ATP demand, the Crabtree effect can be theoretically predicted from ATP yields and reaction stoichiometries to be apparent as a ∆PPR_resp_/∆PPR_glyc_ of 0.18.

Subsequent addition of oligomycin induced a further increase in the glycolytic rate to compensate for loss of respiratory ATP production. The respiratory proton production rate fell from 33 to 13 pmol/min/μg protein, with the decrease representing the portion of O_2_ consumption coupled to ATP synthesis (it probably underestimates this reaction by 7–9% [20]). The remaining rate of respiratory H^+^ production represents O_2_ consumption driving the mitochondrial proton leak. The increase in glycolytic proton production rate (~90 pmol/min/μg protein) was ~4.5-fold greater than the decrease in respiratory proton production rate (20 pmol/min/μg protein); not too far below the theoretical relationship described above. Inverted, it predicts a ∆PPR_glyc_/∆PPR_resp_ of 5.6, assuming (as in the above paragraph) malate-aspartate shuttle activity and no change in ATP demand.

Finally, the addition of 2-deoxyglucose largely (but not completely) abolished glycolytic H^+^ production, without affecting respiratory proton production from respiration driving proton leak (Fig 2C). It is clear that the residual rate of acidification after addition of 2-deoxyglucose is partly caused by respiration (supported largely by endogenous substrates whose oxidation was insensitive to 2-deoxyglucose) driving the mitochondrial proton leak. There was also a small rate attributed to lactate production from glycolysis that was not inhibited acutely by 2-deoxyglucose. Therefore, subtraction of the sum of these rates from the previous rates does not improve the estimate of the glycolytic rates in the earlier parts of the experiment. However, separating out the total respiratory acidification after each addition, as shown in Fig 2C, does allow the absolute glycolytic rates (blue bars) to be interpreted after each addition.

The third problem is whether the rate of acidification after addition of oligomycin, even after correction for respiratory acidification as in Fig 2C, represents the maximum glycolytic capacity. To address this problem consider the bioenergetic reactions running after the addition of oligomycin (Fig 1B). Oligomycin inhibits the mitochondrial F_1_F_O_-ATP synthase, so in the steady state essentially all of the cell’s ATP production must now come from glycolysis. In the presence of glucose and oligomycin, the rate of glycolysis will depend on the rate of ATP demand by protein synthesis, the Na^+^/K^+^-ATPase, and other ATPases. If the sum of their ATP demand is higher than the glycolytic capacity, glycolysis will run at its maximum rate. However, if glycolytic capacity is more than sufficient to satisfy this demand, glycolysis will run at a rate determined by the sum of the ATP-demand reactions, not at the maximum glycolytic capacity. To determine empirically whether glycolysis is running at maximum capacity, ATP demand should be increased experimentally–a lack of glycolytic response would indicate it was running at capacity, but a further increase in glycolytic acidification rate would indicate that it was not.

### Respiratory chain inhibition improves the assessment of maximum glycolytic capacity

To estimate maximum glycolytic capacity, full inhibition of the respiratory chain by addition of rotenone (to inhibit respiratory complex I) plus myxothiazol or antimycin A (to inhibit complex III) is better than addition of oligomycin (to inhibit the mitochondrial F_1_F_O_-ATP synthase), for two reasons. The minor reason is that addition of rotenone plus myxothiazol fully inhibits respiratory acidification, removing the need for any correction of the observed acidification (Fig 1C). The major reason is that inhibition of oxidative phosphorylation by rotenone plus myxothiazol rather than oligomycin allows the uninhibited mitochondrial F_1_F_O_-ATP synthase to run in reverse to maintain mitochondrial protonmotive force, as a potentially powerful additional ATPase in the cell, increasing the total ATP demand over that in the presence of oligomycin (Fig 1C).

**Fig 3:**
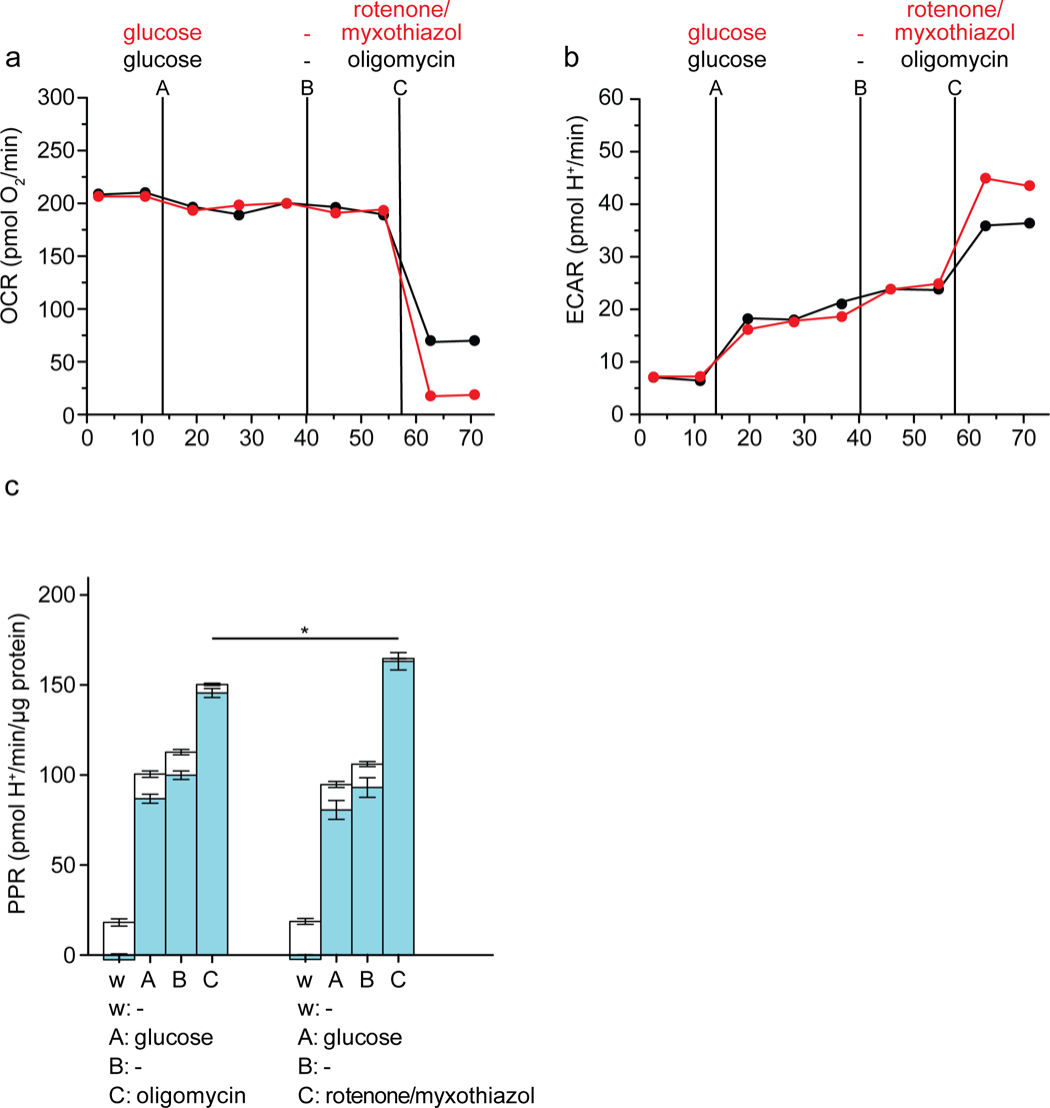
Assay of glycolytic capacity by inhibition of electron transport in C2C12 myoblasts. Raw traces of (*a*) oxygen consumption rate (OCR) and (*b*) extracellular acidification rate (ECAR) after sequential addition using ports A-C of 10 mM glucose, followed by vehicle and then either 2 μg/mL oligomycin (black), or 1 μM rotenone plus 1 μM myxothiazol (red). One representative experiment is shown. *c*: Respiratory (open column sections) and glycolytic (blue column sections) proton production rates (PPR) of the experiment exemplified in a and b calculated using Eq 1. Data are means ± SEM of n = 4 independent biological replicates. *p ≤ 0.05. Statistical analysis was of glycolytic proton production rates only (blue column sections). w, well; A, B, C, addition ports. These data are replotted after Fig 4, where another addition (in port D) is also shown.

Fig 3 compares the apparent maximum glycolytic capacity of C2C12 myoblasts estimated using oligomycin versus rotenone plus myxothiazol. Fig 3A and 3B show the raw data (OCR and ECAR, respectively) and Fig 3C shows the calculated respiratory and glycolytic proton production rates. Use of rotenone plus myxothiazol was clearly superior to addition of oligomycin, since it removed the need for correction for respiratory proton production rate, and gave a significantly higher estimate of maximum glycolytic capacity (Fig 3C). In the presence of rotenone plus myxothiazol and absence of oligomycin, the F_1_F_O_-ATPase hydrolyses ATP from glycolysis to pump protons out of the mitochondria and maintain the protonmotive force against the proton leak that dissipates it, since respiration is inhibited and cannot do so. This will increase the total ATP demand, explaining the increased glycolytic rate observed with rotenone plus myxothiazol compared to oligomycin (Fig 3C).

### Addition of an uncoupler of oxidative phosphorylation does not improve the assessment of maximum glycolytic capacity

**Fig 4:**
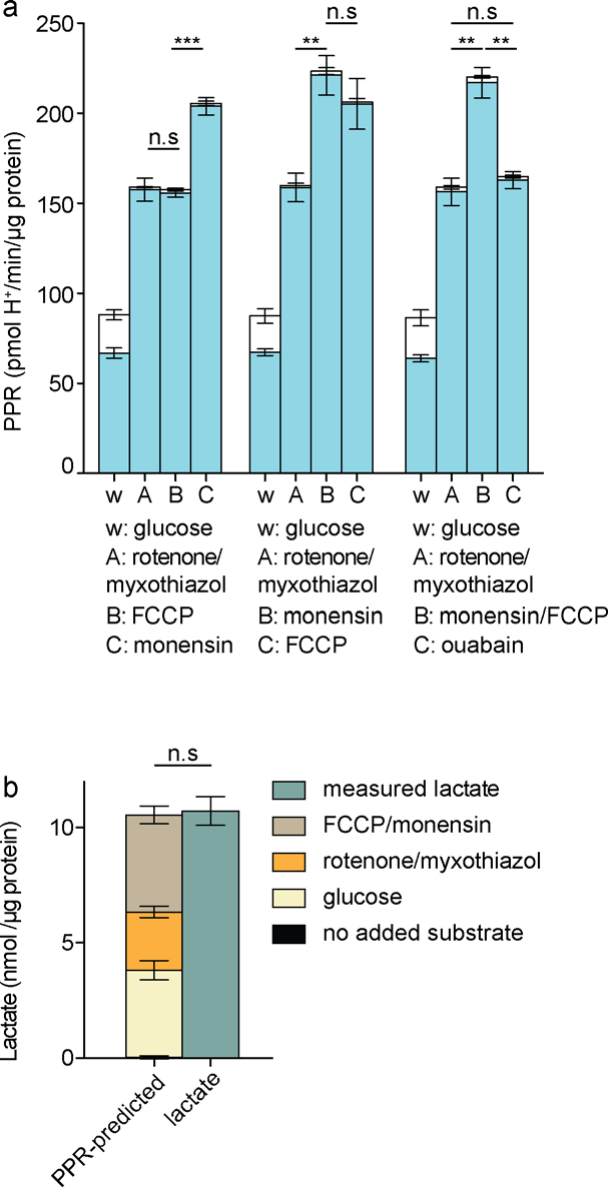
Effects of activating additional ATP consumers on glycolytic rate in C2C12 myoblasts. *a*: Respiratory (open column sections) and glycolytic (blue column sections) proton production rates after sequential additions as shown of 10 mM glucose, 1 μM rotenone plus 1 μM myxothiazol, 200 μM monensin, 1 μM FCCP, and 1 mM ouabain, calculated using Eq 1. Data are means ± SEM of n = 4 independent biological replicates. Statistical analysis was of glycolytic proton production rates only (blue column sections). w, well; A, B, C, addition ports. *b*: Lactate accumulation predicted by glycolytic PPR (left) and measured, in the proposed assay for maximum glycolytic capacity. Data are means ± SEM of n = 3 independent biological replicates. n.s.: not significant; **p ≤ 0.01; ***p ≤ 0.005.

Under these conditions, the rate of ATP hydrolysis by the F_1_F_O_-ATPase will depend on the relatively low endogenous rate of proton leak across the mitochondrial membrane to dissipate protonmotive force. In principle, therefore, increasing the mitochondrial proton conductance by addition of the proton-conducting ionophore FCCP will increase the proton leak rate and increase ATP demand further, allowing the response of glycolytic rate to FCCP to be used to test whether maximum glycolytic rate has been reached, and if not, to increase ATP demand until it is. However, addition of FCCP under these conditions can cause some glycolytic inhibition (5), probably because permeabilization of the plasma membrane to H^+^ allows the plasma membrane potential to drive H^+^ into the cell, acidifying the cytosol by up to 1 pH unit and partially inhibiting glycolysis by changing the kinetics of phosphofructokinase and other pH-sensitive glycolytic enzymes. Fig 4A (left set of bars) shows that addition of FCCP after rotenone plus myxothiazol did not increase glycolytic rate (although addition of another ionophore, monensin, did, see below). We interpret this to mean that FCCP increased ATP demand by the F_1_F_O_-ATPase, but also decreased glycolytic capacity by acidifying the cytosol, so that uncoupling mitochondria by addition of protonophores such as FCCP is unsuitable on its own as a way to establish the maximum glycolytic capacity of cells.

### Activation of the plasma membrane Na^+^/K^+^-ATPase increases glycolytic rate more than respiratory inhibition alone

Another way to increase cellular ATP demand is to increase the rate of the other major ATPase in the cell, the plasma membrane Na^+^/K^+^-ATPase. This can be achieved by adding monensin, an ionophore that exchanges monovalent cations across membranes, primarily extracellular Na^+^ for intracellular H^+^ and K^+^. The influx of Na^+^ raises the cytosolic Na^+^ concentration, causing the Na^+^/K^+^-ATPase to hydrolyse ATP to pump Na^+^ out and restore cytosolic Na^+^. At low monensin concentrations the response of the Na^+^/K^+^-ATPase prevents catastrophic collapse of plasma membrane ion gradients (and consequent inhibition of glycolysis caused by lack of cytosolic K^+^ and slowing of pyruvate kinase) and the Na^+^/K^+^-ATPase increases its ATP demand proportionally to the monensin-catalysed rate of Na^+^ influx.

Fig 4A (middle set of bars) shows that addition of monensin after rotenone plus myxothiazol further increased glycolytic rate, implying that the rate with respiratory inhibitors alone was still submaximal and did not reflect the maximum glycolytic capacity of the cells.

Importantly, addition of FCCP after monensin did not further increase the rate (Fig 4A, middle set of bars). The acidification of the cytosol caused by FCCP addition and the consequent decrease in glycolytic capacity discussed above should be much less marked in the presence of monensin, since H^+^ influx catalysed by FCCP should now be compensated by H^+^ efflux in exchange for Na^+^, catalysed by monensin. This idea is supported by the observation that addition of monensin increased the rate even in the presence of FCCP (Fig 4A, left set of bars), suggesting that monensin was able to largely overcome any decrease in glycolytic capacity caused by FCCP. The increase in rate caused by monensin was fully reversed by the addition of ouabain to inhibit the Na^+^/K^+^-ATPase (Fig 4A, right set of bars), supporting the mechanism outlined above and showing that the effect of monensin was not caused by mitochondrial uncoupling, which would be insensitive to ouabain.

We interpret the lack of stimulation of the rate of glycolysis by FCCP in the presence of rotenone plus myxothiazol and monensin to mean that monensin increased ATP demand sufficiently to reveal the true maximum glycolytic capacity of these C2C12 myoblasts; when ATP demand was further increased by mitochondrial uncoupling to increase the ATP demand by the F_1_F_O_-ATPase under conditions that should largely avoid the secondary decrease in maximum glycolytic capacity, glycolysis could not respond because it was already running at maximum rate. Thus, addition of monensin plus FCCP to C2C12 myoblasts increased ATP demand by the Na^+^/K^+^-ATPase and F_1_F_O_-ATPase sufficiently to exceed the maximum capacity of glycolysis to supply ATP, and addition of monensin plus FCCP is a suitable way to establish the maximum glycolytic capacity of C2C12 myoblasts.

Fig 4A (right set of bars) shows that addition of monensin plus FCCP after rotenone plus myxothiazol increased glycolytic rate significantly compared to addition of rotenone plus myxothiazol alone, defining a suitable protocol for the measurement of maximum glycolytic capacity. The maximum glycolytic capacity measured in this way was about 37% greater than the value estimated by the conventional approach using oligomycin alone, and about 23% greater than the value estimated using rotenone plus myxothiazol alone (Fig 3C). Post-hoc endpoint lactate measurement [5] verified that the proton production rates assigned to glycolysis in this protocol were fully accounted for by lactate production (Fig 4B).

### Assessment of maximum glycolytic capacity when ATP demand is decreased

Immortalized, cultured cells are selected through passaging to grow and divide quickly, making it more likely that they operate at or near metabolic maxima. Addition of oligomycin might therefore elicit glycolytic rates reasonably close to the true maximum capacity of such cells. However, most physiological systems do not operate at respiratory or glycolytic maximum, and we hypothesized that oligomycin would fail even more dramatically to elicit maximum glycolytic capacity when ATP demand was lowered, whereas the improved approach should still work well.

**Fig 5:**
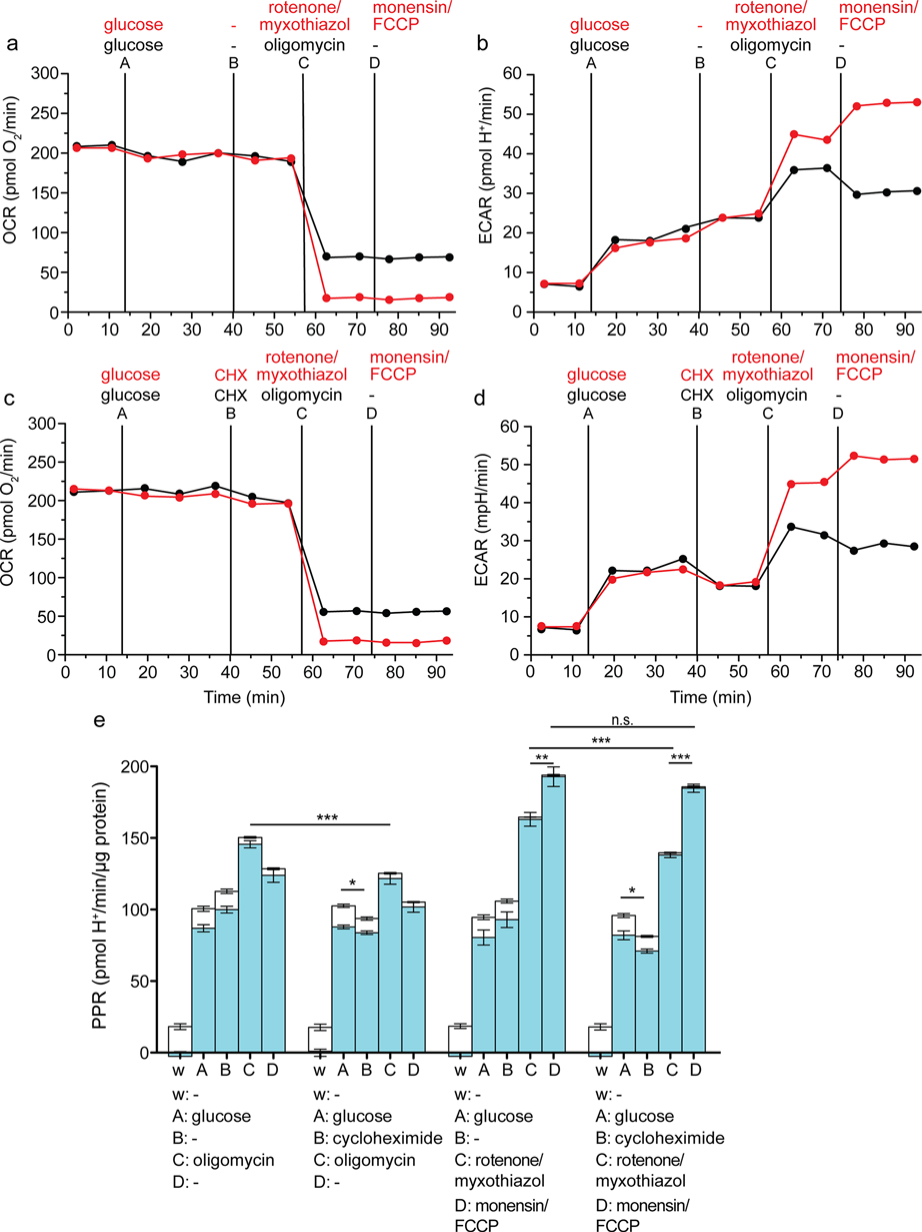
Effects of attenuating ATP demand using cycloheximide on assay of glycolytic capacity in C2C12 myoblasts. Raw traces of oxygen consumption rate (OCR) (*a*, *c*) and extracellular acidification rate (ECAR) (*b*, *d*) after sequential addition using ports A-D of 10 mM glucose, followed by vehicle, and then 2 μg/mL oligomycin (black), or 1 μM rotenone plus 1 μM myxothiazol (red), and then vehicle (black) or 200 μM monensin plus 1 μM FCCP (red). One representative experiment is shown. *e*: Respiratory (open column sections) and glycolytic (blue column sections) proton production rates (PPR) of the experiment exemplified in *a*-*d* calculated using Eq 1. Data are means ± SEM of n = 4 independent biological replicates. n.s.: not significant; *p ≤ 0.05; **p ≤ 0.01; ***p ≤ 0.005. Statistical analysis was of glycolytic proton production rates only (blue column sections). w, well; A, B, C, D, addition ports. A representative raw data file is appended here (S1 Table) with description (S1 Text).

Protein synthesis is a major ATP consumer in cells, particularly (as here) in rapidly proliferating cells, so inhibition of protein synthesis acutely decreases ATP demand to a significant extent [21]. To compare the estimates of maximum glycolytic capacity given by the conventional and improved protocols under different initial states of ATP demand, we added the protein synthesis inhibitor cycloheximide in-flight to the two assay configurations (Fig 1B and 1C). Fig 5A and 5B shows the raw data and Fig 5C shows the calculated contributions of respiratory and glycolytic proton production rates. By decreasing ATP demand, addition of cycloheximide significantly attenuated glycolytic rate (Fig 5E, second and fourth sets of bars).

Because the glycolytic rate after addition of oligomycin does not represent the maximum glycolytic capacity but is limited by ATP demand (see above), the glycolytic rate after addition of oligomycin was significantly less in the presence of cycloheximide (Fig 5E, left two sets of bars, addition C), reinforcing the conclusion that this assay does not measure maximum glycolytic capacity. Similarly, the glycolytic rate after addition of rotenone plus myxothiazol was also significantly less in the presence of cycloheximide (Fig 5E, right two sets of bars, addition C). In contrast, the estimate of maximum glycolytic capacity in the presence of rotenone, myxothiazol, monensin and FCCP in the revised assay was independent of cycloheximide (Fig 5E, right two sets of bars, addition D), reinforcing the conclusion that the revised assay of maximum glycolytic capacity is independent of basal ATP demand. In the presence of cycloheximide, the measured maximum glycolytic capacity with the revised assay was about 52% greater than the estimate from the conventional assay (Fig 5E second set of bars, addition C, and fourth set of bars, addition D).

### Assessment of maximum glycolytic capacity in fibroblasts

**Fig 6:**
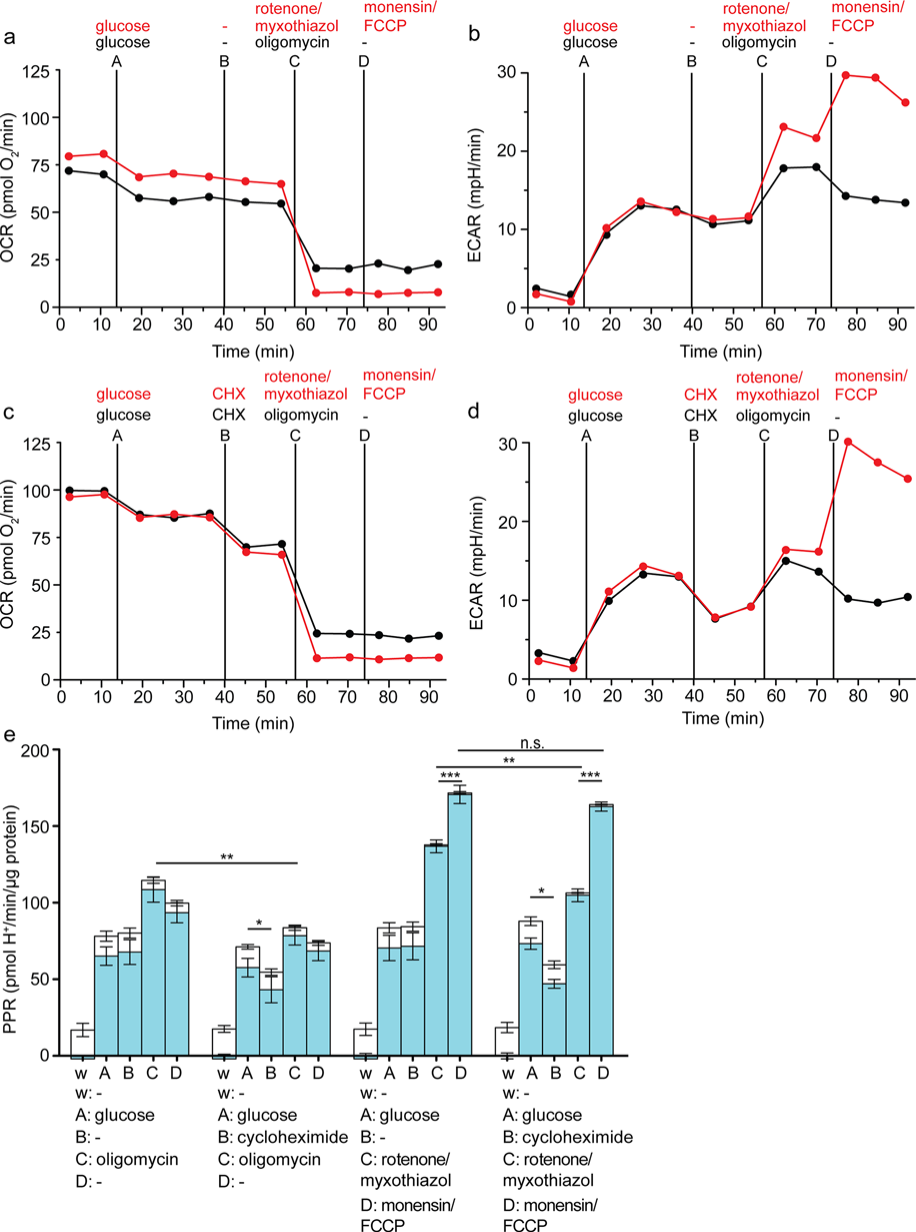
Effects of attenuating ATP demand using cycloheximide on assay of glycolytic capacity in HEK293 fibroblasts. Raw traces of oxygen consumption rate (OCR) (*a*, *c*) and extracellular acidification rate (ECAR) (*b*, *d*) after sequential addition using ports A-D of 10 mM glucose, followed by vehicle or 10 μM cycloheximide (CHX), and then 2 μg/mL oligomycin (black), or 1 μM rotenone plus 1 μM myxothiazol (red), and then vehicle (black) or 200 μM monensin plus 1 μM FCCP (red). One representative experiment is shown. *e*: Respiratory (open column sections) and glycolytic (blue column sections) proton production rates (PPR) of the experiment exemplified in *a*-*d* calculated using Eq 1. Data are means ± SEM of n = 4 independent biological replicates. n.s.: not significant; *p ≤ 0.05; **p ≤ 0.01; ***p ≤ 0.005. Statistical analysis was of glycolytic proton production rates only (blue column sections). w, well; A, B, C, D, addition ports.

Figs 2–5 show data from C2C12 myoblasts, and it is possible that the conditions that elicit the maximum glycolytic rate in these cells do not do so in other cell types that may have insufficient activities of the F_1_F_O_- and Na^+^/K^+^-ATPases. To test this, we repeated the experiments of Fig 5 in HEK293 fibroblasts, with essentially the same results (Fig 6). Cycloheximide significantly lowered the glycolytic rates achieved in the presence of oligomycin or rotenone plus myxothiazol, showing that they were limited by ATP demand, not glycolytic capacity, while it had no significant effect on the maximum glycolytic capacity measured in the presence of rotenone, myxothiazol, monensin and FCCP. The maximum glycolytic capacity measured in the revised assay was significantly higher than the glycolytic rates achieved in the presence of oligomycin or rotenone plus myxothiazol alone, with or without cycloheximide. In the presence of cycloheximide, the measured maximum glycolytic capacity with the revised assay was more than double the estimate from the conventional assay.

### Optimized protocol for assessment of maximum glycolytic capacity

**Fig 7:**
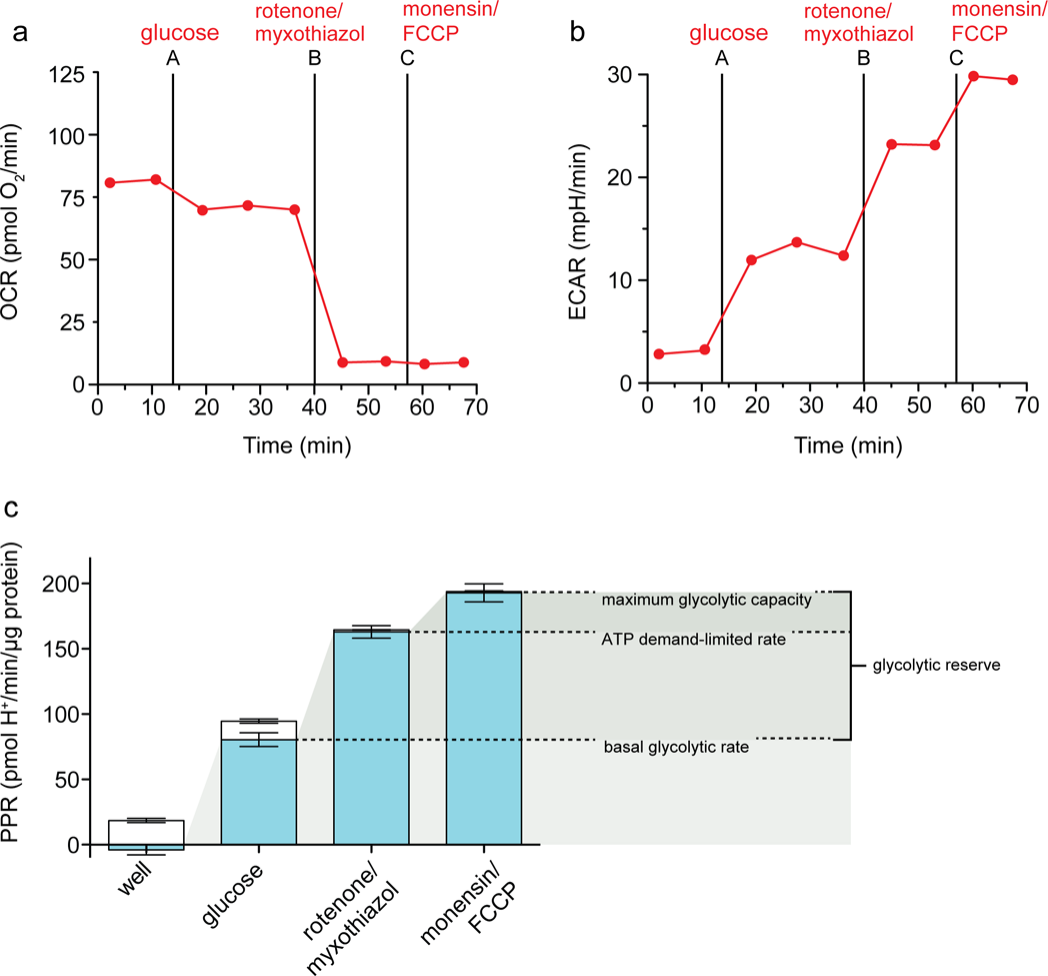
The optimized assay of maximum glycolytic capacity. Raw traces of (*a*) oxygen consumption rate (OCR) and (*b*) extracellular acidification rate (ECAR) in HEK293 fibroblasts after sequential additions of 10 mM glucose, 1 μM rotenone plus 1 μM myxothiazol, and 200 μM myxothiazol plus 1μM FCCP. *c*: Respiratory (open column sections) and glycolytic (blue column sections) proton production rates (PPR) of the experiment exemplified in A and B calculated using Eq 1. Shaded wedges indicate glycolysis under basal conditions (lightest), ATP demand-limited glycolytic rate (medium), and maximum glycolytic capacity (darkest), with the difference between the basal rate and the maximum glycolytic capacity defined as the glycolytic reserve; Data are means ± SEM of n = 4 independent biological replicates.

Fig 7 summarizes the proposed assay (in HEK293 cells) for the measurement of basal glycolytic rate, maximum glycolytic capacity and glycolytic reserve using extracellular flux analysis, incorporating correction of the extracellular acidification rate for respiratory acidification, and using conditions that greatly increase cellular ATP demand to allow better measurement of glycolytic capacity. Fig 7A and 7B show the raw data and Fig 7C shows the calculated and annotated results. Although we show the optimized assay as a three-step assay, it can be run as a two-step assay (additions of (i) glucose and (ii) rotenone, myxothiazol, monensin and FCCP) without losing useful information.

HEK293 cells in the standard minimal assay medium lacking added substrates had undetectable glycolytic rate, but used uncharacterised endogenous respiratory substrates (see Calculations for explanation of assumptions) to provide ATP by oxidative phosphorylation, causing extracellular acidification through CO_2_ production and generation of HCO_3_^-^ plus H^+^. Addition of glucose allowed the cells to switch to mixed ATP production. The rate of oxidative phosphorylation decreased by about 20% as glycolysis took over some of the ATP supply (the Crabtree effect), and the basal glycolytic rate was apparent. Poisoning respiration by the addition of rotenone plus myxothiazol removed the respiratory component of the extracellular acidification rate, caused glycolytic rate to increase to compensate for the lack of oxidative phosphorylation, and caused an additional increase in glycolytic rate to satisfy the increased ATP demand as the mitochondria hydrolysed glycolytic ATP at a relatively low rate to maintain their protonmotive force. Addition of monensin plus FCCP caused a huge increase in ATP demand by the Na^+^/K^+^-ATPase and F_1_F_O_-ATPase, causing glycolysis to run at its maximum rate to try to satisfy that demand, and revealing the maximum glycolytic capacity of the cells. This glycolytic capacity is limited by the concentrations and activities of the metabolite carriers and enzymes making up the glycolytic pathway from extracellular glucose to extracellular lactate, so can reveal changes in these activities independently of ATP demand. The difference between the maximum glycolytic capacity and the basal glycolytic rate can be thought of as the glycolytic reserve that was unused in the basal state but could be recruited in response to increases in ATP demand.

## Discussion

We have demonstrated that respiratory inhibition with oligomycin in the conventional assay for assessing glycolytic rate (Fig 2) is insufficient to elicit maximum glycolytic rate and allow estimation of glycolytic capacity. This is the case even after the contribution of respiratory acidification [5, 22] is recognised and corrected for. The glycolytic capacity assay we propose solves several problems, and we demonstrate its ability to report maximum glycolytic capacity in two different cultured cell lines that are commonly used, under different conditions of cellular ATP demand.

By correcting the total extracellular acidification rate to reveal the underlying glycolytic rate, the true fold increase in glycolytic rate upon addition of glucose is revealed. In C2C12 myotubes or HEK293 cells in the absence of added substrates, all of the extracellular acidification is derived from respiration. Glucose addition induces glycolytic H^+^ production, showing that in the absence of external substrate, these cells utilize endogenous fuels to satisfy all their ATP demands via oxidative phosphorylation, but when glucose is added, they switch to a mixed mode of ATP supply, and glycolytic rate increases by ten- or a hundred-fold or more from near zero.

The increase in glycolytic rate induced by addition of oligomycin can compensate for the loss of oxidative phosphorylation and supply all of the current ATP demands of the cells, but as Figs 3–7 demonstrate, this rate can be less than half of the rate elicited by other compounds, particularly when ATP demand is artificially depressed by inhibition of protein synthesis, and it cannot, therefore, represent the maximum glycolytic rate.

Addition of 2-deoxyglucose does not provide further information about glycolytic rate. As the corrected proton production rate shows, 2-deoxyglucose abolishes much of the glycolytic H^+^ production but does not affect production of CO_2_ and H^+^ from respiration driving the mitochondrial proton leak. To subtract this rate from the total, as conventionally suggested, would therefore cause mis-estimation of glycolytic rate in the preceding measurements. Additionally, our previous work [5] and the quantitative accounting for acidification by respiratory CO_2_ in the absence of added substrates (Figs 2 and 7) suggest that only respiration-derived CO_2_ and glycolysis-derived lactate contribute significantly to extracellular H^+^ flux and that other potential sources of acidification are either balanced within the cell, or are negligible within measurement error in a well-equilibrated system.

Incomplete substrate oxidation will yield different H^+^/glucose ratios depending on the distribution of flux through different available pathways. In cells with a highly active pentose phosphate pathway (PPP), for example, flux through the PPP will generate an extracellular acidification signal that is not accounted for either by respiratory CO_2_ (calculated from OCR) or by lactate production (measured by endpoint lactate concentration). However, this signal can be easily defined by its sensitivity to specific inhibitors of the PPP, e.g., the glucose-6-phosphate dehydrogenase inhibitor 6-aminonicotinamide.

Our proposed assay (addition of glucose followed by addition of rotenone, myxothiazol, monensin and FCCP) optimizes the measurement of maximum glycolytic capacity in three ways (Fig 1C). First, it eliminates ambiguity in the interpretation of the extracellular acidification rate by abolishing respiratory CO_2_-derived acidification (and though we hope users correct their data to determine both sources of acid production, essential for interpreting the change from basal rate to rate with glucose, they would not need a correction for the maximum glycolytic rate). Second, it leaves the ATP synthase active to run in reverse, allowing it to create an ATP sink to maintain mitochondrial protonmotive force. Third, it stimulates considerable additional ATP demand by the monensin-stimulated plasma membrane Na^+^/K^+^-ATPase and the FCCP-stimulated F_1_-F_O_-ATPase, which drives demonstrably higher glycolytic rates than addition of oligomycin or respiratory inhibitors alone.

While isotope tracing is the most accurate way to measure glycolytic flux, it is possible, as described here, to use extracellular acidification measurements to obtain good estimates of both glycolytic flux and glycolytic capacity. We independently validated this method using lactate measurement, as previously described [5] (Fig 4B).

Why is it important to determine maximum glycolytic capacity? Glycolytic capacity provides a quantitative measure of the machinery of glycolysis under a given set of conditions, and is therefore crucial information in understanding how cells may be limited in their energetic responses in pathology and to various micro- and macro-environments and chemical or pharmacological exposures. Multiple recent papers address the measurement of extracellular flux and the maximum capacities of both respiration and glycolysis [5, 6, 15, 22–28]. This attention likely reflects the accessibility, relative simplicity, and wide application of these measurements, as well as a high degree of interest in investigating their biological implications. For these reasons, it is important to understand the measurements and the assumptions behind empirical determination of maximum capacities. When this is done, extracellular flux analysis enables powerful and quantitative statements to be made about the ATP demand of a cell and the pathways of ATP production that satisfy that demand.

## Supporting Information

S1 Table Raw Seahorse dataset of one independent replicate from Fig 5. (XLS)

S1 Text Description of experimental protocol and well assignments for S1 Table. (DOCX)
