# Supplementary material for: Determining Maximum Glycolytic Capacity Using Extracellular Flux Measurements
Source: PLoS One. 2016 Mar 31;11(3):e0152016. doi: 10.1371/journal.pone.0152016 (PMC4816457; doi:10.1371/journal.pone.0152016)
Supplement: S1 Text — (DOCX) [file pone.0152016.s002.docx]

Supplemental Information: Mookerjee, et al., **PONE-D-15-47801**

Supplementary file S1

Raw data from a representative Seahorse assay is provided in .xlsx format. A total of four treatments were made on C2C12 cells grown, prepared, and assayed as described in the Methods, as follows:

|  |  |  |  |  |  | Port injections | | | |
| --- | --- | --- | --- | --- | --- | --- | --- | --- | --- |
|  | Treatment | | | | In wells | A | B | C | D |
| 1 | conventional assay | | | | - | glucose | - | oli | - |
|  |  |  |  |  |  |  |  |  |  |
| 2 | conventional with CHX in port B | | | | - | glucose | CHX | oli | - |
|  |  |  |  |  |  |  |  |  |  |
| 3 | optimized assay | | | | - | glucose | - | rot/myx | FCCP/mon |
|  |  |  |  |  |  |  |  |  |  |
| 4 | optimized assay with CHX in Port B | | | | - | glucose | CHX | rot/myx | FCCP/mon |
|  |  |  |  |  |  |  |  |  |  |

Where CHX: cycloheximide; rot: rotenone; myx: myxothiazol; mon: monensin.

Well assignments were as follows (numbered by treatment):

|  | Treatment | | | | | |
| --- | --- | --- | --- | --- | --- | --- |
| Row/column | 1 | 2 | 3 | 4 | 5 | 6 |
| A | **blank** | 4 | 2 | 3 | 4 | 1 |
| B | 4 | 3 | 1 | **blank** | 3 | 2 |
| C | 1 | 4 | **blank** | 2 | 1 | 4 |
| D | 2 | 3 | 1 | 3 | 2 | **blank** |
